# Irisin protects the substantia nigra dopaminergic neurons in the rat model of Parkinson’s disease

**DOI:** 10.22038/ijbms.2019.33444.7987

**Published:** 2019-07

**Authors:** Sam Zarbakhsh, Manouchehr Safari, Mohammad Reza Aldaghi, Hamid Reza Sameni, Laya Ghahari, Younes Khaleghi Lagmouj, Khojasteh Rahimi Jaberi, Houman Parsaie

**Affiliations:** 1 Research Center of Nervous System Stem Cells, Department of Anatomy, Semnan University of Medical Sciences, Semnan , Iran; 2 Department of Anatomy, AJA University of Medical Sciences, Tehran, Iran

**Keywords:** Irisin, Mesenchymal stem cells, Parkinson’s disease, Substantia nigra, Tunel

## Abstract

**Objective(s):**

Exercise ameliorates the quality of life and reduces the risk of neurological derangements such as Alzheimer’s (AD) and Parkinson’s disease (PD). Irisin is a product of the physical activity and is a circulating hormone that regulates the energy metabolism in the body. In the nervous system, Irisin influences neurogenesis and neural differentiation in mice. We previously demonstrated that co-treatment of bone marrow stem cells (BMSCs) with a neurotrophic factor reduce Parkinson’s symptoms. Our goal in this project was to evaluate whether Irisin with BMSCs can protect the dopaminergic (DA) neurons in PD.

**Materials and Methods:**

35 adult male Wistar rat weighing (200-250 g) were chosen. They were separated into five experimental groups (n=7). To create a Parkinson’s model, intranasal (IN) administration of the MPTP (1-methyl-4-phenyl-1,2,3,6-tetrahydropyridine) was used. The BMSCs (2×106) and Irisin (50 nm/ml) was used for 7 days for treatment after creation of the PD model. After completion of the tests (4 weeks), their brains were used for the TUNEL and immunohistochemical (IHC) assays.

**Results:**

One of the important results of this study was that the Irisin induce BMSCs transport into the injured area of the brain. Co-treatment of the Irisin with BMSCs increased tyrosine hydroxylase-positive neurons (TH+) in substantia nigra (SN) and striatum of the PD mice brain. In this group, the number of TUNEL-positive cells significantly decreased. Behavioral symptoms were better in the combination group and Irisin simultaneously.

**Conclusion:**

Co- treatment of Irisin with BMSCs protects the DA neurons from degeneration and apoptotic process after MPTP injection.

## Introduction

Parkinson’s disease (PD) is a wide-spread and popular neurodegenerative disorder worldwide. Symptoms include slow motion, muscle stiffness, lack of balance, and trembling hands. Usually, 1 to 4 percent of people over the age of 60 are affected (1, 2). Today, the pharmacological treatment using l-3,4-dihydroxyphenylalanine Levodopa (L-dopa) is very common, but over time, the effects of motor improvement are reduced(3). Our previous research showed that the use of stem cells will improve behavior and protect dopaminergic (DA) neurons in substantia nigra pars compacta (SNpc) (1). Therefore, cell therapy can be a novel treatment for neurodegenerative disease. Today, mesenchymal stem cells (MSCs) (4), neural progenitor cells (5), and embryonic stem cells (6) are used to heal and reduce the symptoms of PD in mice. The uses of adult MSCs have advantages over other stem cells. It is easy to obtain and has high pluripotency. There are no ethical problems regarding these cells in contrast to embryonic or fetal stem cells (7). There is little chance for immune rejection of these cells (7). Previous studies have shown that the use of specific growth factors and antioxidants play a key role in protecting the brain (1, 8). One of the hormones that have protective effects on the central nervous system (CNS) neurons is Irisin (9). This hormone is capable of altering metabolism in many organs (10). Irisin is a kind of myokine that increases energy consumption. The Irisin precursor is fibronectin type III domain-containing protein 5 (FNDC5), which is encoded by FNDC5 gene. Irisin is a version of FNDC5 (11). Exercise enhances the activity and expression of some receptors such as peroxisome proliferator-activated receptor gamma coactivator 1 alpha (α). This increased expression results in the production of the FNDC5 protein. Splitting this protein will leads to the production of Irisin (12). Some studies have reported that factors such as Irisin have a neuroprotective effect and can improve the functioning of the nervous system after CNS injury (13). New studies have shown that the Purkinje cells of the cerebellum of rat and mice express Irisin and also FNDC5. Also, mouse embryonic stem cells require FNDC5 to differentiate into neurons (13, 14). Irisin regulates hippocampal neurogenesis by increasing the size and blood flow, changes the morphology of dendrites, increases the synapses, and finally increases neural proliferation (15). Other findings show that Irisin stimulates the secretion of factors in other organs such as muscle, brain and fat tissues. One of the most important mediators released in the brain by Irisin is brain-derived neurotrophic factor (BDNF) (16). BDNF causes brain development by keeping the cells alive, migration and stem cells differentiation, spreading the dendrite and dendritic spine, and finally by synaptogenesis (17). The best pharmacological dose for neural proliferation is 50-100 nm/l (18). This increase seems to happen via the mechanism of signal transducer and activator of transcription (STAT3) but not adenosine 5′monophosphate-activated protein kinase (AMPK) and/or extracellular signal-regulated kinase (ERK) (18). The hippocampus is one of the most important parts that are involved in PD and Alzheimer’s disease (AD) (11). Also in PD, mitochondrial defects and energy are the main causes of the onset of the disease (19), On the other hand, because Irisin regulates energy balance, so there may be a relationship between them.Since there has been no research on the association of PD with Irisin, in this study we evaluated whether Irisin can play an effective role in neurodegenerative disease. 

## Materials and Methods

***Experimental protocol***

Thirty-five adult male Wistar rat weighing (200-250 g) at the beginning of the experiment was provided by the experimental center of Semnan Medical University, Semnan, Iran. All rats were housed at four rats per cage with free access to food and water. The temperature of the storage room was maintained at (20–23 ^°^C) in simulated daylight conditions (12-hr dark and 12-hr light). All stages of testing were carried out in accordance with the Ethics Committee of Semnan Medical University, Semnan. The animals were separated into five experimental groups (n=7). The first group received only culture media as a control. Parkinson’s group received 0.1 mg MPTP (1-methyl-4-phenyl-1,2,3,6-tetrahydropyridine) per nostril. The first treatment or third group received bone marrow stem cells (BMSCs; 2×10^6^) by caudal vein (1 week) after PD. The fourth group treated by Irisin (50 nm/ml) intraperitoneally for 7 days (1 week) after PD. The fifth group received BMSCs (2×10^6^) by caudal vein and Irisin (50 nm/ml) intraperitoneally for 7 days (1 week) after PD. After the completion of the tests (4 weeks), their brains were obtained to carry out the TUNEL and immunohistochemical (IHC) process, including tyrosine hydroxylase (TH) staining. 

***Intranasal injection of MPTP***

The toxin MPTP HCl (Sigma) was injected into the nostril of rats by intranasal (IN) injection according to Prediger *et al*. method (20). Briefly, the rats were first mildly anesthetized by using ketamine and xylazine hydrochloride (75 mg/kg – 15 mg/kg) (Sigma). The 12-mm segment of PE-50 tubing was placed into the nose. The tube was linked to a peristaltic pump. The flow rate was adjusted to 10 ml/min. The MPTP HCl was dissolved in ethanol (10% w/v), then in 0.9% NaCl (saline). It was injected into the nostril for 4 min (1 mg/nostril). In the control group, the only saline was injected. The rest time was 1 min after injection in each nostril to restore their normal respiratory activity. 

***Pole test***

Like other rodents, rats use their own hands for daily activities. In this case, basal ganglia play a very important role (21). Previous studies have shown that the pole test can properly evaluate the activity of basal ganglia and the pathways of the DA system (22). One day before performing the test for matching, animals were introduced to the test environment. The tool consists of a wooden rod 60 cm high, 1 cm in diameter, a wooden bullet with a diameter of 1.5 cm above the bar. This wooden rod is fixed by a solid panel on the floor. The animals were placed on a wooden bullet with their upright. This test requires a good balance. First, the animals should head down and then move from the wooden bar to the ground. In this test, two times are important. First, the time to lower the head, and the time it takes for the animal to reach the ground was determined (locomotors activity time, LAT). The pole test was carried out in two rounds. The first test was started at the beginning of the treatment and the final test was performed after treatment.

The tests were performed at least 3 times, and their meanings were calculated for statistical analysis.

***BMSCs isolation***

BMSCs were separated in sterile condition from hind limb of adult male Wistar rats. Using a 21-gauge syringe, the bone marrow was removed from the thigh and tibia, and inserted into the Hanks Buffer Salt Solution (HBSS). For removing of additional tissues, the cell suspension was passed through a cell strainer filter (100 μm). All the passed cells were centrifuged at 1200 rpm for 10 min and cultured into 25 cm^2^ cell culture flask. Culture medium was Dulbecco’s modified Eagle Medium (DMEM, Invitrogen). Culture medium was supplemented with 10% fetal bovine serum (FBS) (Gibco), 1% (v/v) penicillin/streptomycin (Gibco), and then incubated in a 5% CO_2_ incubator at 37 ^°^C. After three days, the culture medium was replaced, and suspended cells and extra tissues were removed. Stem cells such as the BMSCs were attached to the flask floor. BMSCs attached to the floor of the flask were allowed to multiply to fill 80% of the flask floor. After the third passage, other cells, like fibroblast, and fats were removed and only the bone marrow mesenchymal stem cells were able to reproduce and survive (8, 23).

***Antigen markers***

Identification of stem cell surface antigens to detect MSCs was performed after the end of the third passage by flow cytometry technique. The BMSCs analysis kit was used (according to the manufacturer’s instructions). Briefly, bone marrow cells were harvested from the floor of the flask. The pellets rinsed three times with cold buffer. To separate the cells, cell suspension was passed through the cell strainer (100 µm) and then re-suspended in cold stain buffer to a concentration of 2 × 10^4^ cells/ml. There are many markers for BMSCs identification, but we used positive and negative markers as the classical markers for all stem cells presented by the International Society for Cellular Therapy (ISCT). Negative markers that are specific to hematopoietic stem cells were CD34 and CD45 (hematopoietic markers), and positive markers for BMSCs included CD29, and Cd44 (mesenchymal markers). Cells were directly incubated against monoclonal antibodies (fluorescence-labeled) CD29, CD34, CD44 and CD45 (Sigma). Samples were analyzed using a FACS Calibur flow cytometry machine (24).

***DiI labeling***

CM-DiI (1, 1′-dioctadecyl-3, 3, 3′, 3′-tetramethyl indocarbocyanine perchlorate) is a fluorescent lipophilic cationic indocarbocyanine dye. This material is used for different purposes, such as tracing of injected cells, the fate of cells, and neuronal tracing. CM-DiI is a carbocyanine membrane color that increases the fluorescence by adding lipophilic hydrocarbon chains to the lipid membrane of cells. Due to continuous fluorescence production, this color is used as a suitable color for the recognition of cell structures. The cells that were stained with CM-DiI also showed a strong red signal at 600 nm. Before implanting, BMSCs were labeled with the fluorescent dye CM-DiI (Molecular Probes, Invitrogen, USA). Cells with a concentration of 10^6 ^were incubated in 5 µg DiI for 20 min at 37 ^°^C in a 95% air per 5% CO_2_, and 2×10^6^ cells were injected into the vena caudal. 

***Immunohistochemical and histological study***

IHC study was performed to determine the DA nerve population within SNpc and striatum. Animals were anesthetized with ketamine (100 mg/kg) and xylacin (15 mg/kg). The perfusion process with 4% of the fixative solution of paraformaldehyde (PFA) in 0.1 M phosphate buffer (pH 7.4) was performed through the heart to complete the process of brain fixation. Coronal sections of 6-7 µm were prepared. At first, the brain sections were placed in 10% methanol and H_2_O_2_ for 8 min. After that, the sections were washed 3 times with Tris buffer (pH: 7.4), then laid down in citrate buffer (pH: 7.6) in 98 ^°^C for 11-16 min and rinsed with Tris (pH: 7.4) 3-5 times. The sections were blocked inside the 10% normal goat serum, 0.3% Triton X-100, and 1% bovine serum albumin (BSA), for 2 hr at room temperature (25-27 °C). The sections were placed in the primary antibody (Abcam 6211), which had been solved in 0.3% Tris-buffered saline (TBS) and 1% BSA (1:500) and reacted one night at 4 ^°^C. Afterwards, the sections were washed 4 times every 6 min in the TBS. After this process, the sections were placed in the secondary antibody (FITC-conjugated) (1:200 Abcam 214879), for 2 hr at room temperature (25-27 ^°^C). They were washed in PBS several times at the end. Finally, the sections were stained with DAPI (4, 6-diamidino-2-phenylindole), dehydrated and cover-slipped. The numbers of stain cell bodies were counted. After each 4 sections, one section was counted. Total SNpc and striatum were counted. NIH Image J software was used. After identifying the areas of the striatum and SNpc at low magnification (×4 objectives), to prevent the counting of neurons for a second time, positive cells were counted just when their nuclei were clearly visible (8). 

***TUNEL staining***

The TUNEL test was used for identifying DNA fractures. The method was based on factory instructions (Roche, Basel, Switzerland). In summary, after washing the samples in PBS, they were fixed with 4% PFA for 1 hr. The samples were washed in a solution containing 3% H_2_O_2_ in 70% methanol for 15 min and then rinsed with PBS 3 times. Next, the sections were blocked in 0.1% Triton X- 100 solutions, and 0.1% sodium citrate for 3 min on ice. The sections were then rinsed with PBS. The TUNEL reagent solution (50 μl) was added to each section, and the specimens were incubated for 70 min at 37 ^°^C in the dark. At the end, DAPI (Santa Cruz Biotechnology) was used for counterstaining. The colored cells were evaluated by fluorescence microscope (Olympus).

***Statistical analysis***

Data were described as a mean± standard mean error (SEM). One-way ANOVA followed by Turkey’s *post hoc *test was used to analyze the DA neuron count. A value of *P*<0.05 was considered statistically significant.

## Results

***Flow cytometry***

**Figure 1.**
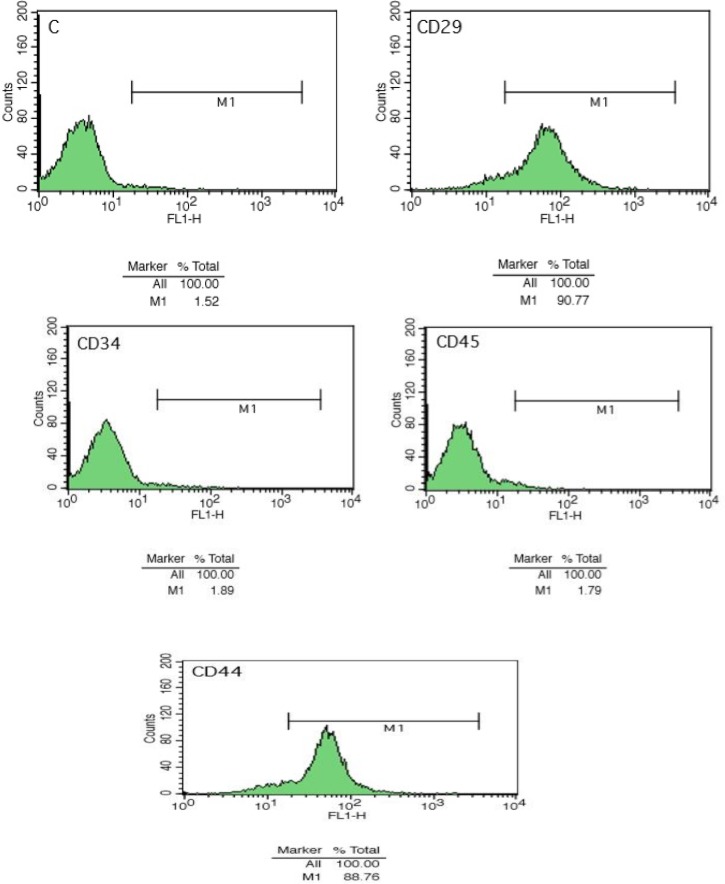
Flow cytometry showed that bone marrow stem cells (BMSCs) were negative for surface expression of CD45 and CD34 and positive for surface expression of CD29 and CD44

Results of flow cytometry after the third passage showed that the separated cells were negative for surface markers of CD45 and CD34, respectively. However, they became positive for surface expression of CD29 and CD 44 (Figure 1). So, the greater cultured cells were MSCs and not hematopoietic stem cells.

***Pole test***

**Figure 2:**
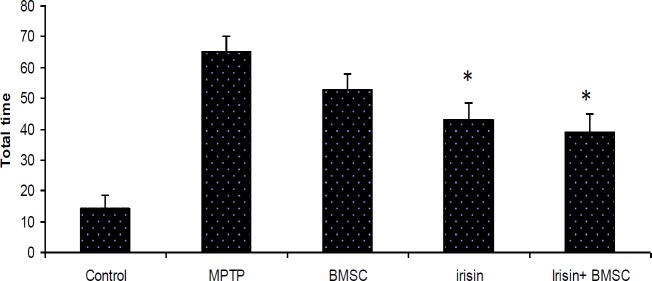
Pole test performance in the experimental groups: The pole test was performed on the 1st week after treatment. The average total time of the three trials was calculated. Total time was not signiﬁcantly affected by the bone marrow stem cells (BMSCs) group compared to MPTP (1-methyl-4-phenyl-1,2,3,6-tetrahydropyridine) (*P*≤0.05). In the Irisin groups alone or plus with BMSCs the differences were significant compared to control and MPTP groups (*P*≤0.05). All values are (mean±SEM). * compared to the MPTP (PD) group (*P*≤0.001)

Results of the locomotors test showed that the total time of descending (TTD) was reduced in the fourth and fifth groups (*P*<0.05) (Figure 2). Total time of descending on MPTP group was (65±12) sec and in the control group was (14±6) sec. Animals could hardly maintain their balance at the top of the rod. In the MPTP group, they were thrown out of the rod during the downfall. The results also indicated that TTD in the Parkinsonian group was significantly higher than the control group (*P*<0.05). In a group that received just BMSCs, the mean TTD was not significantly different with MPTP (53±10 vs 65±12). In the fourth and fifth groups, the difference of TTD was significant in comparison with the PD group (43±5 and 39±6 vs 65±12) (*P*<0.05). No statistical significant difference was observed between the fourth and fifth group (43±5 and 39±6) (*P*<0.01). In all treatment groups, animals could maintain their balance at the top of the rod. Their falling numbers were lowered during the downfall. In the treatment groups with Irisin, most animals used a jumping method to get down from the rod.

***Immunohistochemistry***

**Figure 3:**
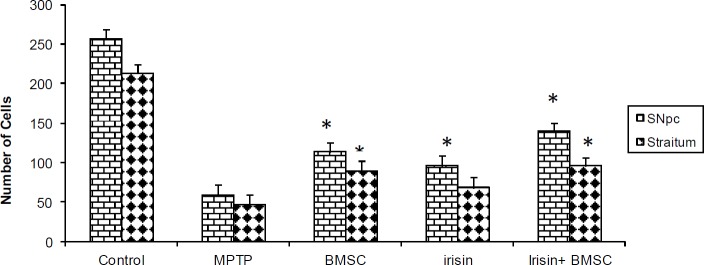
Immunohistochemical analysis of positive tyrosine hydroxylase (TH) cells in the substantia nigra pars compacta (SNpc) and striatum of the brain Rats. There is a significant difference in the average of TH-positive neurons in the SNpc in all treatment groups in comparison with MPTP (1-methyl-4-phenyl-1,2,3,6-tetrahydropyridine) (*P*≤0.05). There is no significant difference in the striatum of the Irisin treatment compared to MPTP. All values are (mean±SEM). * compared to the MPTP group (*P*≤0.001)

**Figure 4:**
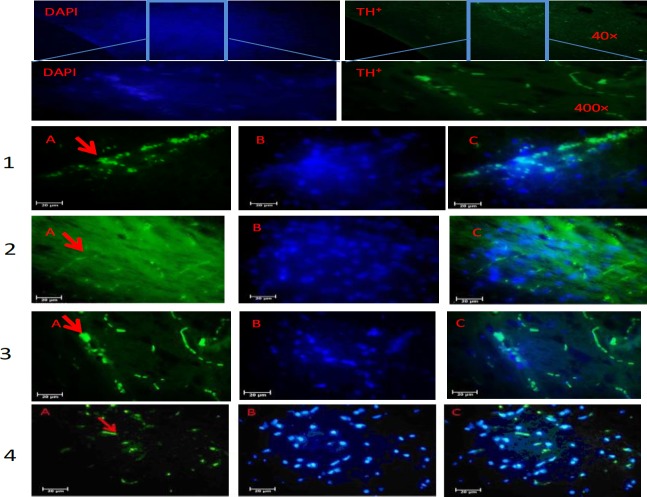
Immunohistochemical staining of tyrosine hydroxylase (TH) positive cells in the substantia nigra pars compacta (SNpc) of the Rats. 1: control, 2; MPTP (1-methyl-4-phenyl-1,2,3,6-tetrahydropyridine), 3: Irisin plus bone marrow stem cells (BMSCs) and 4: BMSCs group. A: primary antibody to TH, B: nuclei stained with DAPI (4, 6-diamidino-2-phenylindole), C: merged. There is a significant difference in the average of TH-positive neurons in the SNpc in all treatment groups in comparison with MPTP (*P*≤0.05). Maximum increase was in the Irisin plus BMSCs group. All values are (mean±SEM). * compared to the MPTP group (*P*≤0.001)

Results showed that MPTP causes significant reduction of TH positive cells in the SNpc, and striatum of the rats. However, treatment with Irisin only or Irisin plus BMSCs significantly protected DA neurons from damage by the MPTP toxin. IHC staining showed that MPTP would significantly reduce DA neurons in the striatum and SNpc region in comparison with control group (59.2±3.2, 46.3±4 vs 256.1±9.5, and 213±6.4 *P*<0.001) as illustrated in (Figure 3 and 4). In the MPTP group, 77% neuronal reductions were observed. So, MPTP has caused the maximum neuronal damage in SNpc. However, treatment with Irisin significantly protected DA neurons from MPTP. Neuronal damage in the SNpc in treatment groups with Irisin was 63%, in BMSCs 56% and in Irisin plus BMSCs was 46%. The number of TH ^+^neurons in SNpc region of the BMSCs and Irisin groups were (113.2±7.2, 95.3±4.9). And in the group of Irisin plus BMSCs, TH^+^ neurons in the striatum and SNpc areas were 139.5±7.3 and 95±4.9 (*P*<0.05). Results also showed that most of the TH^+^ cells were observed in the group of Irisin plus BMSCs. There were a significant difference in the number of TH^+^ neurons in all treatment groups compared to the Parkinsonian group (*P*<0.001). The results confirmed that there is no significant difference between the groups of Irisin and BMSCs (*P*<0.05). TH staining showed the significant preservation of TH positive cells in all treatment groups. Therefore, according to the above results, it can be concluded that Irisin can cause stem cell migration to SNpc and stratum and also convert them into DA neurons. 

***TUNEL staining***

**Figure 5:**
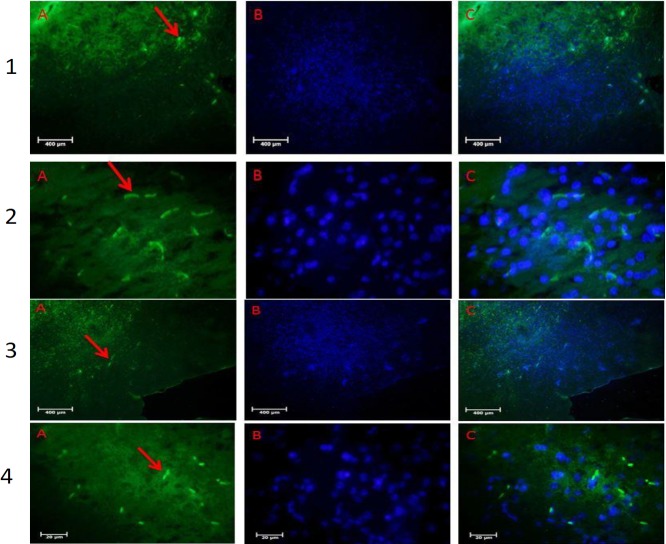
TUNEL staining in the substantia nigra pars compacta (SNpc) of the Rats. 1: control, 2: MPTP (1-methyl-4-phenyl-1,2,3,6-tetrahydropyridine), 3: Irisin plus bone marrow stem cells (BMSCs) and 4: BMSCs group. A: Apoptotic cells, B: nuclei stained with DAPI (4, 6-diamidino-2-phenylindole), C: merged. TUNEL staining showed that the maximum TUNEL positive cells are in MPTP group and significantly decreased when using Irisin plus BMSCs

Other studies have shown that that the TUNEL test is an appropriate test to recognize apoptotic neurons (25). At the end of the treatment, the TUNEL test was performed and the colored neurons were counted (Figure 5). Apoptotic cells were recognized in the SNpc and striatum of all groups. However, TUNEL-positive cells in the MPTP group were more than the other groups. The average number of apoptotic cells in the SNpc area of the control group was (20.62±0.9) and in MPTP was (62.76±1.6). The mean number of apoptotic neurons in MPTP group was more than 3 times that of the control group. The use of Irisin and BMSCs alone or combined with each other could prevent the death of nerve cells and reduce the number of apoptotic cells. The results showed that Irisin plays an important role in reducing the number of apoptotic cells. The number of TUNEL-positive cells in BMSCs group was (46.8±. 8), in Irisin (39.4±1.1) and Irisin plus BMSCs (28.6±1.5). The maximum reduction was observed in Irisin plus BMSCs. There was not a significant difference between Irisin and BMSCs group.

***CM-DiI staining***

**Figure 6:**
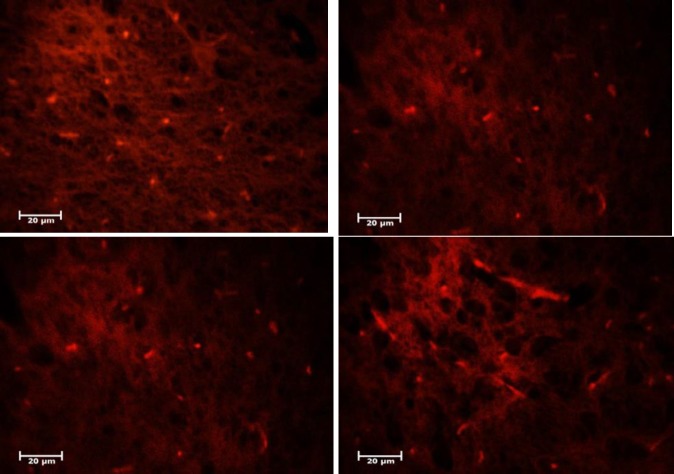
DiI staining revealed bone marrow stem cells (BMSCs) marked cells pass the blood-brain barrier and deployed in the brain. They deployed in injured part of the brain, especially in substantia nigra pars compacta (SNpc) and stratum

CM-DiI labeling was performed to evaluate the placement of injected cells. Since the CM- DiI contains a fluorescence color that is attached to BMSCs, it can show the position and dispersion of the injected cells in the area. The results of histological studies showed that labeled BMSCs were located in the SNpc and striatum of the injured brain (Figure 6). Sections showed some labeled cells located in various sections of the brain. Therefore, injected cells were able to pass the blood-brain barrier and distribute in the different part of the brain including the lesion site. 

## Discussion

In the present study, the results showed that Irisin as a neurotrophic factor protects DA neurons after MPTP treatment. So, Irisin induces migration and differentiation of the exogenous BMSCs into DA neurons in the injured site of the brain.  

Although the function of this molecule is the regulation of the energy metabolisms (11), it also express in different tissues like bones, adipose, and nervous system (26). In the CNS, Irisin causes neurogenesis and differentiation of stem cells into the neuron in the hippocampus (26). Studies have shown that in *fndc5* knockdown mice, the neuronal differentiation and proliferation is impaired (18). According to previous research, neurogenesis is achieved by the STAT3 pathway (18). *Fndc5 *is extremely expressed in the brain. It is necessary for maturation of the new neurons (14). Increasing the Irisin in the blood causes excessive expression of the BDNF in the blood and brain. BDNF is an important key neurotrophic factor that supports the plasticity of the brain (31). BDNF induces the strength of synaptic connections in the brain (27). It can also pass the blood-brain barrier (32). Previous studies have clearly shown that Irisin is a factor that is necessary for the production of the BDNF in the hippocampus (30). Striatal neurons strongly depend on BDNF for performing an activity and survival, which is essentially performed by anterograde transport from corticostriatal afferents (23, 30). The neuroprotective mechanism of BDNF is through the phosphoinositide 3-kinases/ protein kinase B (PI3K/Akt) pathway, which will prevent neural death(4) . Our results showed that if Irisin and BMSCs are used together, the number of TH^+ ^cells in SNpc increased. Therefore, Irisin can induce migration and differentiation of exogenous BMSCs into the lesion site of the brain (SNpc and striatum) in PD. It also caused the number of apoptotic cells in SNpc and striatum in MPTP rats significantly decreased. So, it protects neurons from degeneration and finally causes a recovery. The main role of the exercise is the reduction of chronic inflammation (28). In some neurodegenerative disease like PD or AD, chronic inflammation of the brain is one of the causes of these diseases. Exercise or FNDC5-Irisin significantly reduced the factors of C-reactive protein (CRP), interleukin 6 (IL-6), tumor necrosis factor (TNF), and IL-8 in the blood serum (29). So, Irisin by activating anti-inflammatory routes protects the neurons of CNS in neurodegenerative disorders. 

Previously, it has been shown that exercise has beneficial effects in the human brain and hippocampus, such as increasing blood flow, angiogenesis, increasing dendritic spines, and recovery of synaptic plasticity (33). The *PGC-1 α* gene protects neurons in CNS from the MPTP in mouse (34). Expression of this gene is induced in skeletal muscles with exercise (35) and subsequently in the brain (36). Deficiency of the *PGC-1α* gene in the brain leads to neurodegeneration (37). *PGC-1α* knockout in mice can lead to sever degeneration in the basal ganglia and cortex (36). This phenotype has been considered in PD (30). *PGC-1α* also plays a key role in the maintenance of neuronal function (37). Given the role of this factor in mitochondrial biogenesis, it can act as a neuroprotective agent (34). *PGC-1α* reduces the activity of extrasynaptic receptors of NMDA (N-methyl D-aspartate); therefore, decreases neuronal toxicity in brain neurons of rats, finally protects neurons in the brain (38). Interestingly, administration of Irisin over 7 days may increase *PGC-1 α* expression in different areas of the brain, especially in the striatum and SNpc to protect DA neurons from degeneration. 

## Conclusion

The most important point of this project is that Irisin combined with BMSCs protects DA neurons in SNpc. Also, since Irisin is released after exercise; therefore, exercise can increase the secretion of this factor and prevent PD. Nowadays, the use of pharmacological doses of Irisin can prevent neural damage in the brain, and so prevent from PD.
